# The Resting-State Neural Network of Delay Discounting

**DOI:** 10.3389/fpsyg.2022.828929

**Published:** 2022-03-11

**Authors:** Fan Yang, Xueting Li, Ping Hu

**Affiliations:** Department of Psychology, Renmin University of China, Beijing, China

**Keywords:** delay discounting, fALFF, functional connectivity, the insula, neural network

## Abstract

Delay discounting is a common phenomenon in daily life, which refers to the subjective value of a future reward decreasing as a function of time. Previous studies have identified several cortical regions involved in delay discounting, but the neural network constructed by the cortical regions of delay discounting is less clear. In this study, we employed resting-state functional magnetic resonance imaging (RS-fMRI) to measure the spontaneous neural activity in a large sample of healthy young adults and used the Monetary Choice Questionnaire to directly measure participants’ level of delay discounting. To identify the neural network of delay discounting at rest, we used an individual difference approach to explore brain regions whose spontaneous activities were related to delay discounting across the whole brain. Then, these brain regions served as seeds to identify the neural network of delay discounting. We found that the fractional amplitude of low-frequency fluctuations (fALFF) of the left insula were positively correlated to delay discounting. More importantly, its connectivity to the anterior cingulate cortex was read out for participants’ behavioral performance in the task of delay discounting. In short, our study provides empirical evidence that insula-anterior cingulate cortex connectivity may serve as a part of the neural network for delay discounting.

## Introduction

Life is full of dilemma. Your friend invites you to a big dinner, when you have just decided to lose weight; when you turn on the computer and decide to focus on work, a pop-up window reminds you that the latest episode of your favorite show has just been released. In these situations, you are faced with two options: a small but immediate reward (SIR, the big dinner, and the latest episode) or a large but delayed reward (LDR, the healthier weight, and the work). Although obviously the rational choice is LDR, we often choose SIR instead. This phenomenon is called delay discounting (Kirby and Marakovic, 1995), which refers to the subjective value of a future reward decreasing as a function of time (Kirby et al., 1999). Therefore, if the subjective value of LDR is smaller than SIR, we choose SIR. Delay discounting is found to be associated with many problematic behaviors, such as low academic performance (Kirby et al., 2005), internet addiction (Saville et al., 2010), substance abuse (Kollins, 2003), and pathological gambling (Dixon et al., 2003).

Many studies have been conducted to explore the neural mechanism of delay discounting. Neuroimaging studies have identified several brain regions involved in delay discounting, including the precuneus (McClure et al., 2007; Lim et al., 2017), the dorsolateral prefrontal cortex (Monterosso et al., 2007; Ho et al., 2016), the ventromedial prefrontal cortex (Marco-Pallares et al., 2010; Civai et al., 2016), the insula (McClure et al., 2007; Wittmann et al., 2010; Clewett et al., 2014; Grodin et al., 2017), and the anterior cingulate cortex (ACC) (Monterosso et al., 2007; Hoffman et al., 2008; Ortiz et al., 2015; Grodin et al., 2017; Dennis et al., 2020). Among all these brain areas, the insula plays a critical role in delay discounting through time perception, cognitive control, and emotion processing (Wittmann et al., 2010; Nieuwenhuys, 2012; Li et al., 2013; Noreika et al., 2013; Clewett et al., 2014), whereas the ACC has an effect on delay discounting by participating in the process of conflict detection and strategy adaptation (van Veen et al., 2004; Peters and Buechel, 2011; Li et al., 2013; Wang et al., 2017). Interestingly, both the insula and the ACC are critical nodes of the salience network (SN) identified at resting-state (Seeley et al., 2007; Menon and Uddin, 2010), and the SN is reported to influence delay discounting by playing a significant role in the process of high-level cognitive control (Li et al., 2013; Chen et al., 2017; Grodin et al., 2017; Zhu et al., 2017; Chen et al., 2018). Here, we hypothesized that the resting-state neural network constructed by the insula and the ACC may play an important role in delay discounting.

To do this, we first examined whether the insula or the ACC was associated with delay discounting at resting-state in a large population of participants (*N* = 264). Specifically, we measured fractional amplitude of low-frequency fluctuations (fALFF) of the insula and the ACC, along with the rest of the brain, and then correlated the fALFF values with participants’ behavioral performance in delay discounting. Having established the resting-state neural correlates at the regional level, we further investigated the role of the neural work constructed by these regions in delay discounting.

## Materials and Methods

### Participants

A total of 310 college students (186 women, 18–23 years of age, mean age = 20.36, SD = 0.85, 8 without age information) participated this study. Participants reported no history of neurological or psychiatric disorders. This study was approved by the Institutional Review Board of Beijing Normal University. Prior to the experiment, written informed consent was obtained from all participants.

### Measures of Behaviors

#### Delay Discounting—Monetary Choice Questionnaire

Our study used MCQ (Kirby et al., 1999) to evaluate the degree of delay discounting for each participant. MCQ consists of 27 items, for each item, the participants chose between one SIR and one LDR. For example, “Would you prefer ¥31 today or ¥85 in 7 days?” There was no time limit for the participants to fill in the MCQ. After finishing the questionnaire, each participant received not only monetary compensation but also the reward based on their choice randomly selected from the 27 items to ensure the participants made choices based on their genuine preference. According to the MCQ (Kirby et al., 1999), we calculated *k* values to represent the participants’ degree of delay discounting. The larger the *k* value, the higher the participant’s impulsivity, which means that the participant was more likely to choose SIRs. There are 10 *k* values in the MCQ, each of them had a corresponding choice pattern. The *k* value whose choice pattern was most approximate to the participant’s was the *k* value indicating this participant’s degree of delay discounting. Finally, as the *k* values were not normally distributed, we performed log-transformation to obtain the Ln(*k*) values.

#### Intelligence—Raven’s Advanced Progressive Matrices

Previous studies indicate that there is a significant correlation between intelligence and delay discounting (Shamosh et al., 2008; Shamosh and Gray, 2008). Therefore, we included intelligence as a confounding factor which was indicated by Raven score (Raven et al., 1998). Raven’s Advanced Progressive Matrices contains 36 items, requiring the participants to select the missing figure to complete a 3×3 matrix. The number of correct answers in 30 min was taken as the Raven score of each participant.

### Image Acquisition

Resting-state functional magnetic resonance imaging scanning was conducted on a 3T Siemens Trio scanner (MAGENTOM Trio, a Tim system) with a 12-channel phase-arrayed coil at Beijing Normal University Imaging Center for Brain Research, Beijing, China. During the resting-state scan, participants were instructed to keep still, remain awake, close their eyes, and not purposely think of anything. The RS-fMRI scanning consisted of 240 contiguous echo-planar imaging (EPI) volumes (TR = 2000 ms; TE = 30 ms; flip angle = 90°; number of slices = 33; matrix = 64×64; FOV = 200 × 200 mm^2^; acquisition voxel size = 3.125 × 3.125 × 3.6 mm^3^). Additionally, high-resolution T1-weighted images were obtained with a magnetization-prepared gradient echo sequence (MPRAGE: TR/TE/TI = 2530/3.39/1100 ms; flip angle = 7°; matrix = 256×256; number of slices = 128; voxel size = 1 × 1 × 1.33 mm^3^) for spatial registration.

### Image Data Preprocessing

Resting-state functional magnetic resonance imaging image data were preprocessed with FSL (FMRIB Software Library^1^). The preprocessing steps included head motion correction (by aligning each volume to the middle volume of the 4-D image with MCFLIRT), spatial Gaussian smoothing (FWHM = 6 mm), realignment, intensity normalization, and the removal of linear trends. To better eliminate the influences of psychological noise on our subsequent analysis, we regressed out 18 nuisance signals from cerebrospinal fluid, white matter, global brain average, and motion correction parameters. The registration of each participant’s RS-fMRI to the anatomical images was accomplished by using FMRIB’s Liner Image Registration Tool (FLIRT) (Jenkinson and Smith, 2001; Jenkinson et al., 2002) to produce a six degree-of-freedom affine transformation matrix. The registration of each participant’s anatomical images to the Montreal Neurological Institute (MNI) space was carried out by using FLIRT to calculate a 12 degree-of-freedom linear affine matrix. Because low-frequency fluctuations are sensitive to brain activity in gray matter, we defined a gray mask with the possibility threshold of 0.5 in SPM8. In total, 128,190 voxels were included in the gray mask.

### Statistical Analysis

Since 38 participants had missing scan data, 6 participants had missing age or gender information, and 2 participants’ Raven scores were outside ±3 standard deviations, 264 participants (159 women, 18–23 years of age, mean age = 21 years, SD = 2.12) were included in the following analyses. The kurtosis and skewness of age (0.05, −0.21) and Raven scores (−0.12, −0.30) were within the range from −1 to +1, which indicates the normality of age and intelligence (Marcoulides and Hershberger, 1997).

#### Fractional Amplitude of Low-Frequency Fluctuations-Delay Discounting Correlation Analysis

To explore the relationship between delay discounting and spontaneous neural activities, we calculated the correlation between the Ln(*k*) values and the fALFF values of each voxel across the whole brain, with age, gender, intelligence, and head motion parameter as the confounding factors. According to Zou et al. (2008) and Zuo et al. (2010), each participant’s fALFF value of each voxel was obtained through dividing the sum of amplitudes across the entire frequency range (0–0.25 Hz) by a fractional sum of the amplitudes within the low-frequency range (0.01–0.1 Hz). Multiple comparison correction was conducted by Gaussian random field theory (GRF) in the DPABI (Yan et al., 2016). The threshold at the voxel level was *p* < 0.05 and at the cluster level was *p* < 0.05.

#### Resting-State Functional Connectivity-Delay Discounting Correlation Analysis

Based on the results from the fALFF-delay discounting correlation analysis, our study examined the neural network of delay discounting at resting-state by using resting-state functional connectivity (RSFC). Firstly, we used the clusters obtained in the fALFF-delay discounting correlation analysis as the seeds. And we calculated the mean time series from all voxels in each seed for each participant. Secondly, for each participant, the RSFC of each voxel was defined as the correlation on the mean time series between the seed and other voxels. Voxel by voxel, the RSFC was calculated across the whole brain, with age, gender, intelligence, and head motion parameter as the confounding factors. Finally, for each seed, we calculated the correlation between the RSFC of each voxel and the Ln(*k*) values to identify the brain network involved in delay discounting. We transformed *r* maps to *T*-score maps. Multiple comparison correction was conducted by GRF in the DPABI (Yan et al., 2016) for the correlations between the RSFC of each voxel to the insula and the Ln(*k*) values. The threshold at the voxel level was *p* < 0.05 and at the cluster level was *p* < 0.05.

## Results

The degree of delay discounting for each participant was indexed by the Ln(*k*) value. A higher Ln(*k*) suggests a greater discount on future rewards, so individuals with higher Ln(*k*) likely prefer immediate rewards (i.e., SIRs). The kurtosis (−0.05) and skewness (−0.04) of participants’ delay discounting were within the range from −1 to +1, which indicates the normality of the Ln(*k*) values (Marcoulides and Hershberger, 1997). The mean of Ln(*k*) values was −5.18 and the standard deviation was 1.54, which suggested that the variance of the Ln(*k*) values was suitable for the individual difference approach to explore the neural correlates of delay discounting.

To exhaustively investigate the brain regions which were associated with delay discounting at resting-state besides the regions of interest (i.e., the insula and the ACC), we correlated the fALFF values of each voxel with the Ln(*k*) values across the whole brain of each participant. After controlling for age, gender, and head motion, we found that the Ln(*k*) values showed a significant positive correlation with the fALFF values in the left insula (cluster size: 684; MNI coordinate: −38, −18, 18; GRF corrected, *p* < 0.05). This result confirmed our hypothesis on the role of the insula in delay discounting, as participants with a larger fALFF value in the left insula may discount the future value more steeply, and thus prefer to choose SIRs. In this analysis, no other significant results, including the ACC, were found.

**FIGURE 1:**
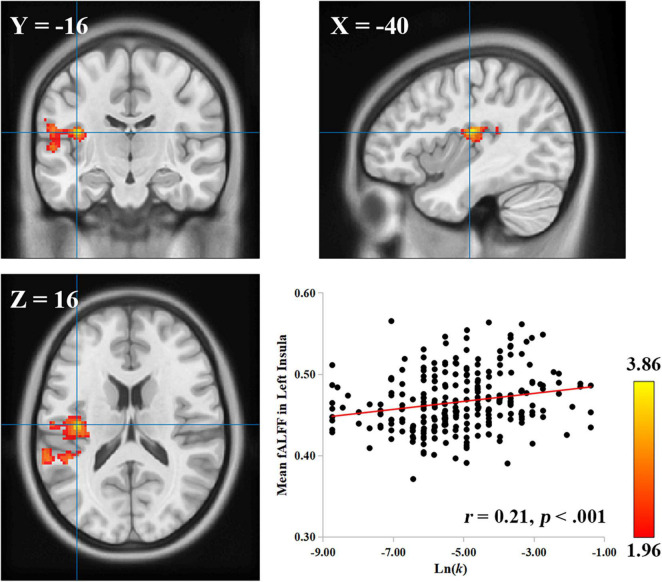
The left insula is related to delay discounting. A cluster with a positive correlation between the fALFF value and the Ln(*k*) was found in the left insula. The scatter plot between the distributions of the Ln(*k*) and the mean fALFF values of the cluster after controlling for intelligence, age, gender, and head motion is shown for display purposes. Each dot represents one participant.

Because the insula is a region with multiple functions—previous studies have shown that the insula is involved in intelligence as well (Takeuchi et al., 2011; Cox et al., 2019), to rule out the possibility that the observed correlation between the left insula and delay discounting may be accounted for by individual differences in intelligence, we conducted a control analysis with intelligence as a confounding factor. The results showed that the correlation between the fALFF values in the left insula and Ln(*k*) values was still significant after controlling for intelligence, age, gender, and head motion (cluster size: 928; MNI coordinate: −40, −16, 16; GRF corrected, *p* < 0.05; Figure 1). In short, the relationship between the left insula and delay discounting at resting-state was stable, unlikely ascribed to intelligence.

**FIGURE 2:**
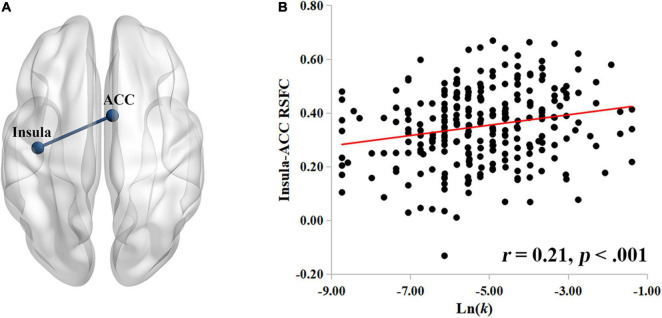
The insula-ACC RSFC is related to delay discounting. **(A)** The insula-ACC connectivity significantly correlated with the Ln(*k*) values. **(B)** The scatter plot showing the positive correlation between the insula-ACC RSFC and the Ln(*k*) values. Nodes and edges in the networks are overlaid on inflated surface maps generated by BrainNet Viewer (Xia et al., 2013).

Having identified the role of the insula during rest in delay discounting, we use the left insula as the seed ROI to construct a neural network of delay discounting with whole-brain resting-state functional connectivity (RSFC). Specifically, we correlated the RSFC of each voxel to the insula with the Ln(*k*) values across the whole brain of each participant. After controlling for intelligence, age, gender, and head motion, we found that the RSFC between the insula and the ACC was significantly positively correlated with participants’ Ln(*k*) values (*r* = 0.21, *p* < 0.001; Figure 2; the ACC cluster size: 979; MNI coordinate: 6, 4, 36; GRF corrected, *p* < 0.05); showing a stronger insula-ACC RSFC corresponded to a higher Ln(*k*) value in the task of delay discounting. That is, participants with a stronger connectivity between the insula and the ACC may discount the future value more steeply, and thus prefer to choose SIRs.

## Discussion

In the current study, we used RS-fMRI to explore the resting-state neural network of delay discounting in a large sample of healthy young adults. First, we found a positive correlation between the Ln(*k*) values and the fALFF values of the left insula, revealing that individuals with larger intensity of spontaneous neural activities in the insula discounted future rewards more sharply, and were more likely to choose SIRs over LDRs. Second, we used the delay discounting-related insula as a seed to construct the neural network for delay discounting, and found that the Ln(*k*) values were positively associated with the strength of the insula-ACC RSFC, suggesting the stronger the connectivity between the insula and the ACC, the more the participants preferred SIR. It is important to note that as the individual difference approach was adopted, the neural network identified in our study reflected the individual differences in delay discounting, which suggests a role for neural networks in other aspects of delay discounting. The insula and the ACC are important nodes of the salience network, and therefore our study illustrates how the salience network plays a pivotal role in delay discounting at resting-state.

In methodology, our study supplements previous studies on the resting-state neural network in three ways. First, instead of pre-selecting regions of interest to construct the neural network of delay discounting as previous studies did (Schmaal et al., 2012; Dias et al., 2013; Contreras-Rodriguez et al., 2015; Rosch et al., 2018; Holmes et al., 2020), here with a large sample of participants we had sufficient power to perform a whole-brain analysis to thoroughly explore the neural correlates of delay discounting. Second, rather than indirectly measuring delay discounting with variables affecting delay discounting (Guo and Feng, 2015; Xin et al., 2020; Zhang et al., 2021), here we used the Monetary Choice Questionnaire to directly calculate Ln(*k*) as an index for delay discounting. Finally, we tested heathy adult participants, which can extend the findings from previous studies based on mental health patients and adolescents (Dias et al., 2013; Contreras-Rodriguez et al., 2015; Wang et al., 2017; Zhu et al., 2017; Rosch et al., 2018; Holmes et al., 2020) to a larger population.

Our novel finding of the insula’s role in delay discounting during rest was in accordance with previous studies using task-based fMRI on healthy participants or patients (Wittmann et al., 2007; Hoffman et al., 2008; Wittmann et al., 2010; Avsar et al., 2013; Lim et al., 2017; Miranda-Olivos et al., 2021). Specifically, our finding that lower intensity of spontaneous neural activities was correlated with the preference of LDRs perfectly echoes the finding from a lesion study, where patients with damaged insulas preferred to choose LDRs (Sellitto et al., 2016). This finding also supports the hypothesis that the insula may modulate delay discounting by controlling the impulsivity of choosing immediate rewards (Menon and Uddin, 2010), not the hypothesis of uncertainty of future rewards (Paulus et al., 2003). Indeed, individuals with high intensity of resting fluctuations at rest may overshadow the neural activity in a task, which makes the inhibition of impulsivity more difficult. Without sufficient cognitive control, individuals may thus prefer immediate rewards.

The resting neural network constructed with the insula serving as a seed also showed correlations with delay discounting. Importantly, only the connectivity between the insula and the ACC reached significance. The insula and the ACC are two key nodes of the salience network (SN), consistent with previous studies showing that the SN is involved in delay discounting through the processing of high-level cognitive control (Li et al., 2013; Chen et al., 2017, 2018; Grodin et al., 2017; Zhu et al., 2017). The SN is a large network consisting of multiple cortical and subcortical regions (Seeley et al., 2007; Menon and Uddin, 2010). Our study illuminates the role of the insula and the ACC of the SN in delay discounting, which advises future studies to narrow down the scope of the interaction of these two regions in delay discounting. It is interesting to note that the insula and ACC are considered to be domain-general regions involved in a variety of processes, such as emotion (Ernst et al., 2014; Gasquoine, 2014; Nelson et al., 2015), autonomic functions (Cechetto, 2014; Rolls, 2016; Roquet and Conti, 2021), and self-referential processing (Northoff et al., 2006; Hu et al., 2016; Yoon et al., 2019). Our finding of the insula-ACC network involved in delay discounting is not contradictory to this mainstream perspective. First, our study did not support the exclusivity of the insula and ACC in delay discounting, because in the study we only ruled out the possible confounding factor of general intelligence in delay discounting. Second, delay discounting is a complex phenomenon, which consists of multiple cognitive components such as cognitive control and reward evaluation (Peters and Buechel, 2011; Miedl et al., 2012; Stanger et al., 2013; Frost and McNaughton, 2017; Patros et al., 2018). These cognitive components are likely recruited for other tasks as well. Therefore, the network identified in this study was only specific but not exclusive to delay discounting. Moreover, note that our network analysis was restricted to the seed of the insula, which was localized by its relevance to behavioral performance of delay discounting with the measure of fALFF. Therefore, we certainly missed abundant neural correlates of delay discounting, some of which may lie in the SN. Future studies shall use pre-defined SN nodes, such as the anterior insula, amygdala, ventral striatum, and hypothalamus, to investigate their relationship to delay discounting. Besides, previous studies have also reported that the interaction during rest between the SN with other large-scale neural networks (e.g., executive control network, default mode network, and frontoparietal network) is also associated with delay discounting (Li et al., 2013; Zhu et al., 2017; Chen et al., 2018). In this study, we tested a large sample of participants and performed a whole-brain analysis, but we failed to find the association between cross-network resting-state connectivity and behavioral performance in delay discounting. One possibility is that here we only explored the network centering in the insula, which might miss significant contributions from connectivity not consisting of the insula. However, we believe this is unlikely given the critical role of the insula in delay discounting, the statistical power of this study, and the direct measure of delay discounting. Future studies with more dedicate designs are needed to examine the cross-network functional connectivity in delay discounting.

In sum, our study employed RS-fMRI to demonstrate the neural correlates in the resting state of delay discounting. We first correlated the fALFF values with participants’ behavioral performance in delay discounting and established that the spontaneous activities in the insula were related to delay discounting at the regional level. We further used the delay discounting-related insula as a seed to investigate the neural work in delay discounting. Our study gives new empirical evidence that as the key nodes of the SN, the connectivity between the insula and the ACC is involved in delay discounting. An important but unaddressed issue is the cognitive components underlying delay discounting. Delay discounting consists of a variety of cognitive components, such as reward valuation, time perception, conflict detection, and cognitive control (Wittmann and Paulus, 2008; Claus et al., 2011; Frost and McNaughton, 2017; Dennis et al., 2020; Lukinova and Erlich, 2021). However, the behavioral index of the delay discounting used in our study (*k* value) was not able to be further decomposed into these cognitive components. On the other hand, the neural correlates of delay discounting may shed light on the cognitive components of delay discounting. For example, previous studies have shown that the ACC is an important node in the reward circuit (Breiter and Rosen, 1999; Haber and Knutson, 2010; Liu et al., 2016); therefore, the insula-ACC connectivity for delay discounting suggests the overlap between the SN and the reward circuit. That is, the function of reward evaluation may underlie delay discounting, which needs to be examined in future studies.

## Data Availability Statement

The raw data supporting the conclusions of this article will be made available by the authors, without undue reservation.

## Ethics Statement

The studies involving human participants were reviewed and approved by Institutional Review Board of Beijing Normal University, Beijing, China. The patients/participants provided their written informed consent to participate in this study.

## Author Contributions

XL and PH designed the study. XL ran the experiments. FY analyzed the data. All authors wrote the manuscript, contributed to the article, and approved the submitted version.

## Conflict of Interest

The authors declare that the research was conducted in the absence of any commercial or financial relationships that could be construed as a potential conflict of interest.

## Publisher’s Note

All claims expressed in this article are solely those of the authors and do not necessarily represent those of their affiliated organizations, or those of the publisher, the editors and the reviewers. Any product that may be evaluated in this article, or claim that may be made by its manufacturer, is not guaranteed or endorsed by the publisher.

## Footnotes

1
www.fmrib.ox.ac.uk/fsl/
